# Yeast biotechnology: teaching the old dog new tricks

**DOI:** 10.1186/1475-2859-13-34

**Published:** 2014-03-06

**Authors:** Diethard Mattanovich, Michael Sauer, Brigitte Gasser

**Affiliations:** 1 Department of Biotechnology, University of Natural Resources and Life Sciences, Muthgasse 18, 1190 Vienna, Austria; 2 Austrian Centre of Industrial Biotechnology (ACIB GmbH), Vienna, Austria

**Keywords:** *Saccharomyces cerevisiae*, Non-conventional yeasts, Metabolic engineering, Biofuels, Recombinant protein, Whole cell biocatalysis

## Abstract

Yeasts are regarded as the first microorganisms used by humans to process food and alcoholic beverages. The technology developed out of these ancient processes has been the basis for modern industrial biotechnology. Yeast biotechnology has gained great interest again in the last decades. Joining the potentials of genomics, metabolic engineering, systems and synthetic biology enables the production of numerous valuable products of primary and secondary metabolism, technical enzymes and biopharmaceutical proteins. An overview of emerging and established substrates and products of yeast biotechnology is provided and discussed in the light of the recent literature.

## Background

The use of yeast for food processing and fermentation of alcoholic beverages is traditionally marked as the primary inventive step of biotechnology, dating back several millennia. The discovery of microbial metabolic activities from the 19th century onward initiated the target-oriented development of yeast bioprocesses which were the prototype of modern biotechnological processes. A major hallmark was the development of the “Zulaufverfahren” for efficient baker’s yeast production [1]. Today this process is employed under the term “fed-batch” to avoid overflow metabolism in the majority of industrial bioproductions. Efficient fermentative processes developed for ethanol production served as a model for the production of different metabolic products from yeasts, filamentous fungi and bacteria.

Bacteria and filamentous fungi have taken over the lead role in the development of bioprocesses around mid of the 20th century [2]. However since then novel developments of recombinant protein production, metabolic engineering, and systems and synthetic biology, paired by the demand for many products which can be synthesized by yeasts enable a plethora of new applications of yeasts in biotechnology. We see three major fields of application for yeasts in modern biotechnology: production of metabolites, production of recombinant proteins, and *in vivo* biotransformations.

Traditionally “yeast” denotes *Saccharomyces cerevisiae* and its close relatives, used for alcoholic fermentation and baking. However today about 1500 yeast species have been identified (a variable number due to current reclassifications). Biotechnologists have summarized all non-*S. cerevisiae* yeasts which they use as “non-conventional” yeasts. What unifies them is a lower degree of fermentative overflow metabolism [3] and a rather short history of genetic and biological characterization. The lifestyle of *S. cerevisiae* is characterized by flourishing in extremely high sugar concentrations – disposing most of it as the fermentative by-product ethanol. Most natural habitats however do not provide such extreme substrate conditions so that most non-conventional yeasts provide alternative metabolic routes for substrate utilization and product formation, and different regulatory patterns. A few species of major interest are *Pichia pastoris* (syn. *Komagataella pastoris*), *Hansenula polymorpha* (syn. *Ogataea parapolymorpha*), *Yarrowia lipolytica*, *Pichia stipitis* (syn. *Scheffersomyces stipitis*), or *Kluyveromyces marxianus*.

The classical carbon substrates for yeast processes are glucose or sucrose, derived mainly from corn starch and cane sugar. Extrapolating the successful expansion of industrial biotechnology, and most importantly considering the food requirements of mankind lets us envisage a shortage of these classical substrates, driving research towards the utilization of alternative carbon sources. Lignocellulose hydrolysate constitutes such an abundant carbon source, requiring yeasts that can utilize xylose and arabinose (the major constituents of hemicellulose). These are either natural pentose assimilating yeasts like e.g. *Pichia stipitis*[4] or *Hansenula polymorpha*[5], or *S. cerevisiae* strains with engineered pentose utilization pathways [6]. Alternatively, glycerol as an abundant by-product of biodiesel production is explored as a substrate for yeast processes. While also *S. cerevisiae* can utilize glycerol, the uptake and assimilation is much higher in other yeasts like *Pachysolen tannophilus*[7], *Y. lipolytica*[8,9], or *P. pastoris*[10].

**Figure 1:**
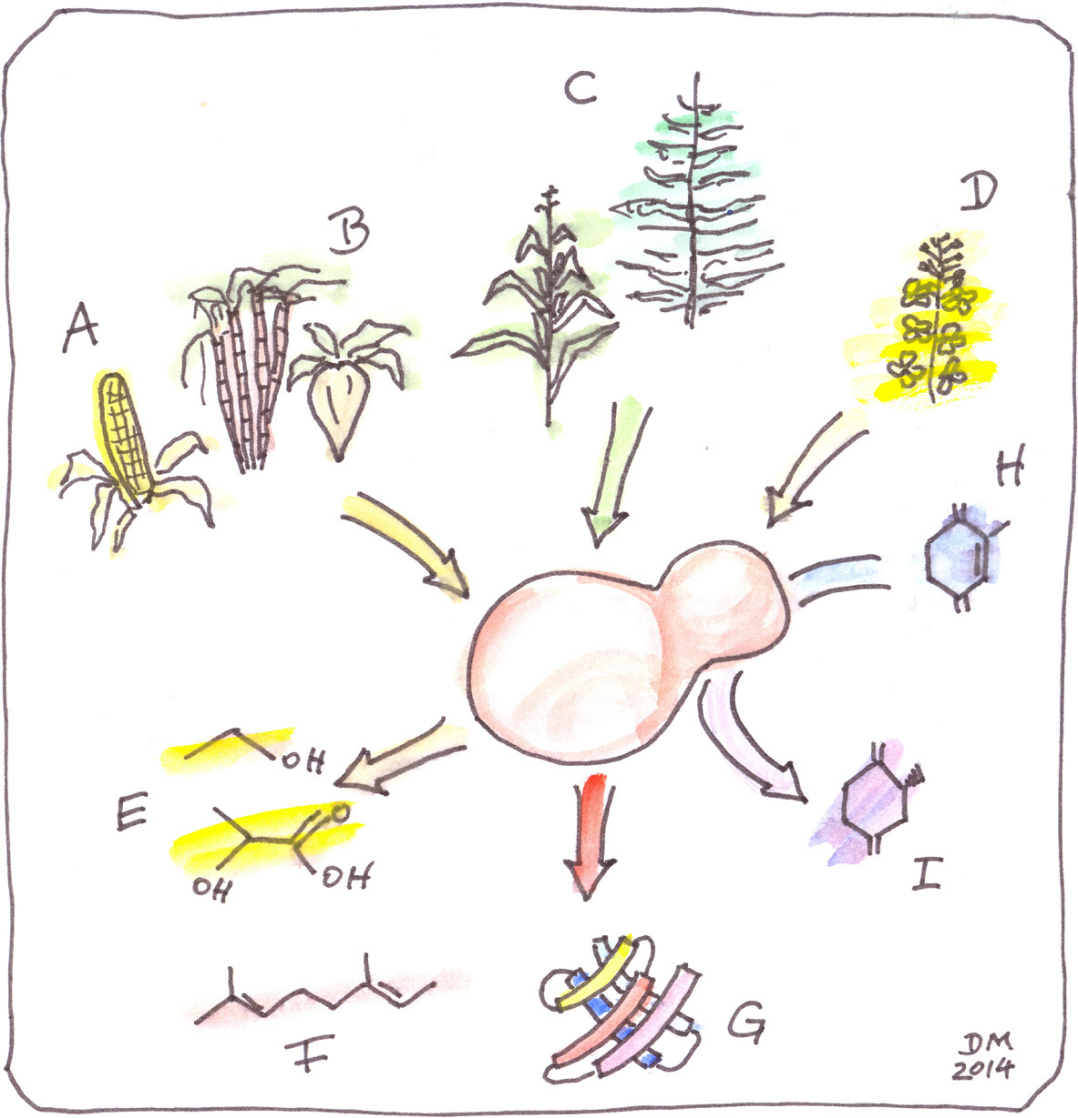
**Substrates and products of yeast bioprocesses.** Main carbon sources employed in yeast bioprocesses are derived from **(A)** corn starch, **(B)** cane or beet sugar, **(C)** lignocellulose (corn stover, straw, wood etc.) and (D) crude glycerol from biodiesel production. Different native and engineered yeast strains convert the substrate to products of **(E)** primary or **(F)** secondary metabolism, or **(G)** recombinant proteins. Whole cell biocatalysis is a special case where **(H)** a complex substrate is biochemically transformed to **(I)** a product by the metabolic activity of yeast cells. Chemical structures are illustrative images only.

Different substrates and products of yeast biotechnology are summarized in Figure 1. In the following, the main current applications of yeasts are discussed.

### Yeasts as platforms for metabolite production

Due to the foreseeable limitation of mineral oil resources the interest in biotechnological production of chemicals by microbial metabolic activities is ever increasing. While the first wave of metabolite production processes used natural producers of desired molecules (e.g. production of astaxanthin by *Phaffia rhodozyma* (syn. *Xanthophyllomyces dendrorhous*) or production of riboflavin by *Pichia guilliermondii*), current concepts aim at engineering a few platform strains for the production of many chemicals. Several physiological features predestine yeasts as such a platform [11]: high substrate uptake rates, potentially high metabolic rates, robustness against stressful process conditions. Additionally, there is also a revival in engineering of natural production hosts for improved productivity as the increasing availability of yeast genome sequences enables better understanding and relief of rate-limiting steps based on the results of Omics data and metabolic modelling.

Ethanol made by yeast is by far the largest biotech product [12]. The development of more efficient and robust strains using different substrates has been a major driving force for the development of the yeast platform. However, ethanol as a biofuel suffers from a low substrate yield and a rather low energy content, so that the production of other alcohols like butanol or isobutanol is attempted today [13-16].

Several short chain organic acids are valued precursor chemicals. Production of the free acid requires low pH of the culture broth, so that the acid tolerance of yeasts is a valuable feature. Low pH lactic acid production has been achieved in *S. cerevisiae*[17], *P. stipitis*[18], *Candida boidinii*[19] and *Candida sorenensis*[20] and is reaching industrial scale. Succinic acid production with engineered *S. cerevisiae* is employed in a process announced to reach 30,000 t/y scale in 2015 [21].

Efficient pathways for production of phenolic substances such as flavonoids [22] and stilbenoids [23] have been developed but still need further increase of productivity and yield.

Isoprenoids are a universal class of molecules all based on the same building blocks. This universality enables to design novel pathways in yeasts, using the native core structures with specific conversions carried out by heterologous pathways. Thereby, often recombinant genes from different species are combined to obtain the intended variety. Isoprenoids encompass more than 40,000 plant secondary metabolites, a number of them with pharmaceutical activity. Recently yeast based production of the antimalaria agent artemisinin reached commercial production [24]. Isoprenoids produced with recombinant yeast have also been proposed as biobased jet fuel [25].

Polyketides are complex biomolecules mainly of bacterial or fungal origin. Recombinant expression of polyketide synthases in yeasts enables the study of their complex function [26] and the development of heterologous production strains [27,28]. The heterologous production of synthetic penicillins in yeasts has been suggested as well [29].

### Recombinant protein production in yeasts

*S. cerevisiae* has been the first yeast employed for production of heterologous proteins [30]. In the early 1980s this was the only yeast species with significant molecular genetic characterization which explains its wide commercial use in the following years for production of human insulin and hepatitis B surface antigen. It has turned out however that other yeasts are more efficient in the production of many recombinant proteins [31-33]. A current literature survey indicates that most work on recombinant protein production in yeasts is performed with *P. pastoris* and *H. polymorpha*, followed by *S. cerevisiae* and *Y. lipolytica*. In 2009, about 20% of the biopharmaceutical products approved in U.S.A. and Europe were produced in *S. cerevisiae*[34]. Other yeast platforms play an important role in clinical studies and have begun to enter the biopharma market in the recent years.

Secretion of recombinant proteins to the culture supernatant constitutes a major bottleneck of yeast production hosts [35], favouring some non-conventional yeasts over *S. cerevisiae*[32,33]. A genomic comparison of the secretory pathway of 8 yeast species indicates that *S. cerevisiae* and its close relatives have lost some functions of secretory protein quality control [36]. Engineering of folding and secretion related genes is a valuable strategy to enhance the secretory capacity of yeasts [35,37-39], however the comparison to mammalian cells like Chinese hamster ovary cells shows that there is still a lot of room for improvement [40].

Systems biology has strongly contributed to our current understanding of limitations of protein production [41]. Genome scale transcriptomics and proteomics revealed physiological reactions to protein overproduction [42]. Overexpression has a severe impact on primary metabolism reflecting a higher demand for energy and reducing equivalents [43,44] and free amino acids [45,46]. Metabolic engineering may further channel the flux towards required precursers (own unpublished data), and may also contribute to enhanced protein secretion by providing sufficient cofactors, e.g. heme [47].

### Whole cell biocatalysis

To differentiate from microbial metabolite production, whole cell biocatalysis may be defined as the conversion of organic compounds by enzymatic activities of life cells. The main advantages compared to classical biocatalysis are the cheap production of the required enzymatic setup, and/or the use of the cellular metabolism for cofactor regeneration. Recombinant yeast whole cell biocatalysts have been developed for the conversion of cephalosporins [48] and steroids [49], and the asymmetric reduction of α-keto esters [50]. Whole cell biocatalysts usually exert their activity intracellularly. However also secreted enzymes may act in *in vivo* biotransformations. E.g. D-tagatose has been produced from D-lactose by secretory production of bacterial β-D-galactosidase and L-arabinose isomerase [51]. Yeast surface display of lipase enabled the whole cell based production of phospholipids and fatty acid methyl esters [52].

## Conclusions

Recent research has generated exciting new developments of products and bioprocesses using yeasts. Common patterns of successful yeast based processes are the efficient use of substrate, and a closed energy and redox balance where metabolic engineering may serve to meet the extra demand of product formation. Engineering the protein secretory pathway solves specific problems in overproduction of recombinant proteins.

To provide a forum for scientific discourse, Microbial Cell Factories has initiated a thematic series on Yeast Biotechnology [53]. This virtual series will continue to compile the most relevant papers in yeast research published in Microbial Cell Factories, to serve research in this field for the benefit of mankind.

## Competing interests

The authors declare that they have no competing interests.

## Authors’ contributions

All authors have contributed equally to this commentary, and all have read and approved the final manuscript.
